# Tunneling spectroscopy of close-spaced dangling-bond pairs in Si(001):H

**DOI:** 10.1038/srep14496

**Published:** 2015-09-25

**Authors:** Mads Engelund, Rafał Zuzak, Szymon Godlewski, Marek Kolmer, Thomas Frederiksen, Aran García-Lekue, Daniel Sánchez-Portal, Marek Szymonski

**Affiliations:** 1 Centro de Física de Materiales CSIC-UPV/EHU, Paseo Manual de Lardizabal 5, E-20018, Donostia-San Sebastián, Spain; 2 Center for Nanometer-Scale Science and Advanced Materials, NANOSAM, Faculty of Physics, Astronomy and Applied Computer Science, Jagiellonian University, PL-30-348, Krakow, Poland; 3 Donostia International Physics Center, Paseo Manual de Lardizabal 4, 20018, Donostia-San Sebastián, Spain; 4 IKERBASQUE, Basque Foundation for Science, E-48013, Bilbao, Spain

## Abstract

We present a combined experimental and theoretical study of the electronic properties of close-spaced dangling-bond (DB) pairs in a hydrogen-passivated Si(001):H p-doped surface. Two types of DB pairs are considered, called “cross” and “line” structures. Our scanning tunneling spectroscopy (STS) data show that, although the spectra taken over different DBs in each pair exhibit a remarkable resemblance, they appear shifted by a constant energy that depends on the DB-pair type. This spontaneous asymmetry persists after repeated STS measurements. By comparison with density functional theory (DFT) calculations, we demonstrate that the magnitude of this shift and the relative position of the STS peaks can be explained by distinct charge states for each DB in the pair. We also explain how the charge state is modified by the presence of the scanning tunneling microscopy (STM) tip and the applied bias. Our results indicate that, using the STM tip, it is possible to control the charge state of individual DBs in complex structures, even if they are in close proximity. This observation might have important consequences for the design of electronic circuits and logic gates based on DBs in passivated silicon surfaces.

The miniaturization of electronic devices has pushed fabrication techniques towards the atomic scale. The invention of the scanning tunneling microscope (STM) made the characterization of structural and electronic properties of such devices possible^1^. This is very important for the future realization of atomic or monomolecular electronic devices^2^,^3^.

Hydrogen-passivated IV-semiconductor surfaces are attracting growing attention as promising substrates for utilization in nanoscale circuits^4^,^5^,^6^,^7^,^8^,^9^,^10^, because they allow creation of surface dangling bonds (DBs), which introduce new electronic states within the intrinsic band gap of the hydrogenated surface^10^,^11^. This opens up new possibilities to modify the electronic properties at the atomic scale, as well as to create atomic-scale circuitry and devices.

In recent years it has been shown that DBs can be fabricated with atomic precision by local desorption of hydrogen atoms using the STM tip^10^,^11^,^12^,^13^. To this day, numerous application ideas have been developed and tested, including the implementation of qubits^10^, the fabrication of locally doped wires and their application in prototypical nanoscale transistors and other electronic circuits^5^,^6^,^14^,^15^, as well as the creation of surface electronic/logic circuits based on the quantum Hamiltonian computing approach^16^,^17^,^18^. It has been shown that the charge state of a single, isolated DB is highly dependent on substrate doping^19^ and effects induced by the proximity of a STM tip^4^,^20^. In particular, Bellec *et al.*^8^ have even demonstrated controlled reversible switching between charge states using an STM tip. It remains an open question how these effects are modified by the interaction between two or more DBs.

In this paper we study close-spaced DB pairs (*i.e.*, DBs located in nearest-neighbor Si dimers) created with atomic precision on a moderately p-doped silicon substrate. Scanning tunneling spectroscopy (STS) data are acquired at cryogenic temperatures and show a non-trivial asymmetry depending on the DB site within the pair (see Fig. 1). We consider two different types of DB pairs, called “cross” and “line” structures. The STS spectra taken on different DBs of the pair are almost identical, except for a constant energy shift whose magnitude depends on the pair type. This is to the best of our knowledge the first focused study of pairs of nearest-neighbor DBs. Schofield *et al.*^20^ reported STS measurements for different DB structures in n-doped Si(001):H. However, in their structures there was always at least one hydrogen-passivated surface Si atom between each DB of the DB pairs considered. STS data are also available for the very closest DB pair - unsaturated Si dimers in Si(001):H^11^,^21^. These DB-dimers behave quite distinctly from the pairs of the more separate DBs considered here. First, they exhibit a very different electronic structure, with DB-derived states much closer to the edges of valence and conduction bands. Second, they undergo high frequency fluctuations during STM imaging, a feature which is not observed for the systems studied here. For these reasons, in the present study we leave aside DB dimers and focus here on pairs of more separate DBs, systems which have received little attention to date.

Here we propose that, in order to understand the persistent, but moderate asymmetry of the experimental *dI*/*dV* curves, it is necessary to invoke two ingredients: (i) the ground state of the formed DB pairs in our p-doped substrate being inherently asymmetric, with one of the DB always positively charged; (ii) the easy transition of the charge state of those positive DBs to a neutral charge state under the influence of the proximity of the STM tip.

## Methods

### Experimental setup and procedure

The experiment was carried out in an ultra-high vacuum (UHV) system manufactured by Omicron Nanotechnology GmbH. The samples, provided by CEA-LETI, Grenoble, were prepared in a clean room as two reconstructed Si(001):H surfaces, bonded face to face by dispersive forces^12^. This method enables transportation of the bonded wafers in ambient conditions without any damage to the reconstructed Si(001):H surfaces. After insertion into the UHV system the samples were debonded using a home-built device and subsequently STM measurements were performed at liquid helium temperature (4.5 K). SPIP and WsxM^22^ software was used for data processing and analysis.

The used Si(001):H samples had a nominal 10^15^–10^16^ cm^−3^ p-doping level, and a very low density of defects. In Fig. 2A the typical filled state image of the Si(001):H surface is shown with clearly visible reconstruction rows running along the 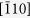 crystallographic direction. The preparation of the DB structures was performed following the protocol of Kolmer *et al.*^12^. The tip was placed over the hydrogen atom assigned for desorption. Then, the feedback loop was switched off and the sample voltage was ramped up to around 3.5 V. The desorption of the hydrogen atom was observed as a sudden increase in the tunneling current. After desorption of the first hydrogen atom, the tip was placed over the next hydrogen atom designated for removal.

**Figure 1:**
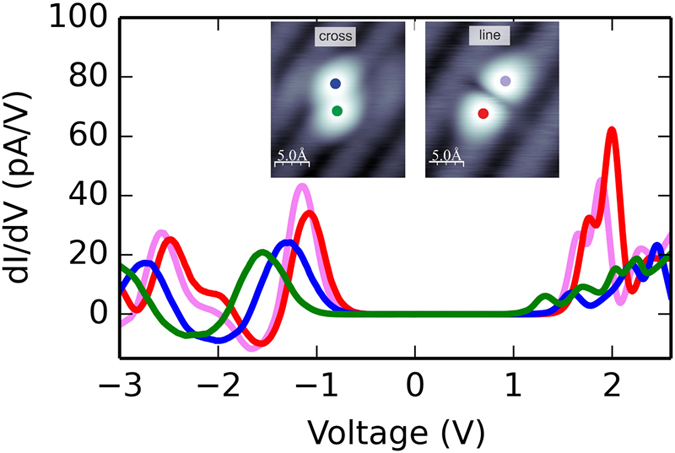
Filled-state images and STS acquired over each site of the two types of DB pairs. Insets show filled-state STM images of the “cross” and “line” structures, respectively. Colored dots indicate the position of the tip during STS recording (sample voltage −2.0 V, tunneling current 10 pA) and the STS data from that position are plotted in the same color.

**Figure 2:**
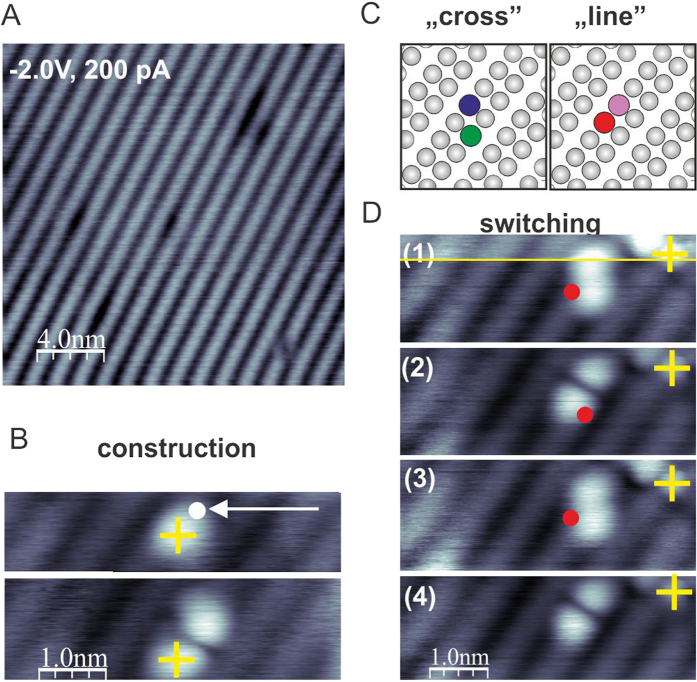
DB structures formed with the STM tip. (**A**) Filled-state STM image of the Si(001):H sample acquired after debonding of the wafer in UHV system. (**B**) Series of two filled-state STM images showing the construction of the DB pair. The upper panel shows a single DB, while the lower panel contains a DB pair. The white dot indicates the position of the tip during desorption of the second hydrogen atom and the yellow mark is a fixed position. (**C**) Structural models of the “cross” and “line” structures, gray balls mark the hydrogen atoms, colored balls indicate individual DBs. (**D**) Series of filled state STM images presenting the reversible switching between “cross” and “line” structures, red dots indicate the position of the tip during switching event. All STM images in B and D were acquired with a sample voltage of −2.0 V and a tunneling current of 10 pA. The yellow line in D1 indicates the scan line at which the scanning was paused, the position of the DB closer to the red dot was switched, and the imagining was later continued.

The desorption procedure was then repeated leading to a pair of DBs located in neighboring Si surface dimers. Figure 2B details the construction of the structure showing both the initial DB site and the completed DB pair. Two different types of pairs were investigated, the “line” and “cross” pairs (Fig. 2C). Note that both DB pairs are located within the same dimer row of the reconstructed surface. Interestingly, it was possible to switch between the “line” and the “cross” using the STM tip. The switching occurs when the sample voltage is raised to the moderate value of 2.5 V being low enough to prevent the desorption event. Figure 2D presents a series of switching events between the two structures. After creating the DB pair, the electronic properties were probed using STS techniques (see Fig. 1). For each pair, the STS spectra taken over both DBs seem to be a shift of an otherwise generic spectrum. For the “cross” pair this relative shift is ~0.3 V, while for the “line” pair the smaller shift of 0.07 V is accompanied by a change in relative peak heights. These shifts were reproducibly recorded for both pair structures.

The acquisition of the STS data was performed in spectroscopy mode where the feedback loop was switched on between two consecutive STS measurements to establish the tip height at the desired position (feedback loop setpoint: −2.0 V, 10 pA). Then the feedback loop was switched off and the current versus voltage (*I*–*V*) curve was recorded. The plotted *dI*/*dV* were obtained by numerical differentiation of the collected *I*–*V* characteristics. To increase the quality of the data, the STS measurement over the assigned DB was repeated 50 times and the resulting *I*–*V* traces were averaged before the differentiation was performed. The *I*–*V* curves exhibit a clear asymmetry between the two sites of the symmetrically constructed structure, in particular a clear shift of the peak positions. The entire procedure of DB preparation and STS measurement was repeated to confirm that the observed site asymmetries were indeed reproducible.

### Calculational scheme

**Figure 3:**
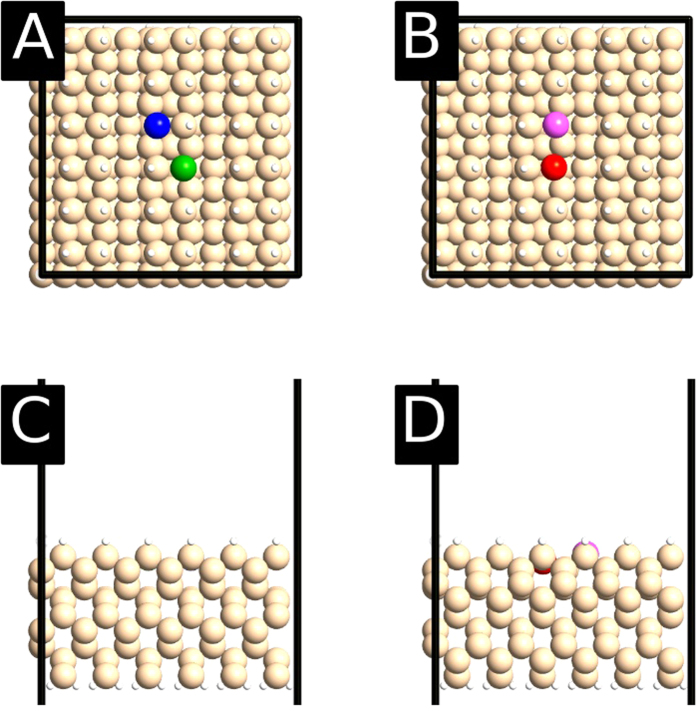
Examples of relaxed geometries of the neutral “cross” (left) and “line” (right) DB pairs in Si(001):H. Top panels show a top view of our simulation cell, while a lateral view is shown in the lower panels. White and peach color balls represent hydrogen and silicon atoms, respectively. The “cross” DB sites are shown in blue and green, while the “line” DB sites are shown in red and purple. For the “cross” a symmetric configuration is shown while an asymmetric one is shown for the “line” (0.66 Å height difference between the two Si atoms at the DB sites). In this neutral charge state it was possible to stabilize both symmetric and asymmetric neutral structures for both types of DB pairs, but in both cases the symmetric configuration had the lowest energy.

In order to investigate the systematic asymmetry between the two sites of the DB pairs we have performed spin-polarized density-functional theory (DFT) calculations based on the SIESTA code^23^,^24^,^25^. As shown in Fig. 3, the surface was represented by a Si slab containing 9 layers. The top surface is covered by the Si(001)-(2 × 1):H dimer reconstruction with two hydrogen vacancies, while the bottom surface was fully passivated with hydrogen. We used periodic boundary conditions with a 6 × 6 supercell of the unreconstructed Si(001) surface cell, yielding a total of 430 atoms. We used the generalized gradient approximation (GGA) to describe exchange and correlation^26^, a *k*-point sampling of 2 × 2 × 1, a mesh-cutoff of 200 Ry for the real-space integrations, and a double-*ζ* plus polarization (DZP) basis set. Relaxations were performed until all forces were below 0.02 eV/Å, fixing the coordinates of the lower 4 Si layers in their bulk positions and the bottom passivating hydrogen layer fixed in previously relaxed positions. Population analysis was performed by integrating the electron density inside Voronoi polyhedra^27^ around each atom using a fine real-space grid corresponding to a plane-wave cutoff of 500 Ry.

According to our interpretation the key ingredient to explain the observed asymmetry of the STS spectra is the influence of the STM tip and the applied bias on the charge state of the DB pairs on the surface. The different charge states were simulated by supplying a net charge of 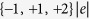 to the system by adding or subtracting electrons, along with a compensating homogeneous background. Since the unoccupied DB states are located fully within the gap, all free charge goes to the DB and the systems were relaxed with this extra free charge. Additionally, two meta-stable neutral configurations were relaxed.

Note that with our charging model it is only possible to compare the stability of configurations with the same background charge. We can, however, compare the positions of energy levels by using a common reference of energy. In our calculations, the lower passivating hydrogen layer provides this common reference, more precisely, the centroid of the projected density of states (PDOS) over the occupied states.

## Results and Discussion

At first, the observed asymmetry of the STS spectra might not seem very surprising. Both in the case of the single unsaturated Si-Si dimer and the fully unpassivated Si(001) surface, the Si dimers at the surface are buckled at low temperature^28^,^29^. One could similarly expect nearest-neighbor DB pairs to suffer a structural distortion, accompanied by a charge redistribution, that would make the two DBs inequivalent^30^. This is indeed confirmed by our DFT-GGA calculations. For both “cross” and “line” DB pairs it is possible to stabilize asymmetric as well as symmetric geometries. Relative energies are shown in Table 1. For both pairs the most stable structure is symmetric, although energy differences are rather small, particularly for the “line” configuration. Figure 3 shows two examples of such structures (the symmetric one for the “cross” DB-pair and the asymmetric one for the “line” DB-pair). The structural distortion gives rise to one doubly occupied DB gap state and one empty state roughly associated with each of the DB sites. As observed in Table 2, the asymmetric geometries are matched by asymmetric charge distributions within the pair, with DB sites above (below) the height of the neutral DB accumulating (losing) electron charge.

One could think that such asymmetric configurations would capture the essential physics of the experimentally observed asymmetry. In such case, one would expect a very asymmetric STS spectrum with respect to the bias polarity, *i.e.*, strong (weak) signals at positive (negative) bias at the one DB, and the inverse behavior at the other DB. Surprisingly, this is not what is observed in Fig. 1. Thus, rather than the asymmetry, what appears to be counterintuitive is that the STS curves taken over different DBs within each pair are *almost* identical, essentially only disturbed by a relatively small energy shift. Note that for the “cross” pair both DBs show peaks of similar heights at negative bias, which are only slightly shifted in energy. This cannot be explained by a spontaneous symmetry breaking and tunneling into a static asymmetric electronic or geometric structure.

In order to clarify the observed behavior we propose that it is necessary to take into account the influence of the proximity of the STM tip and the applied bias on the charge state of each DB, which is modified as the voltage is ramped to acquire the *dI*/*dV* curves. The possibility to control the charge state of isolated DBs in Si(001):H has been demonstrated in several recent works^4^,^8^. In our interpretation of the present STS data the DB site being measured (*i.e.*, the one closest to the STM tip) changes its charge state as electrons tunnel on and off the site, while the other DB site (not being measured) remains basically unmodified. With this assumption we are able to explain the measured data as detailed below, including the origin and the size of the observed shifts.

We first need to pinpoint the relevant charge states for our model. We will do so by comparing the experimental STS spectra with the PDOS onto the Si atoms at the DB sites for each of the calculated charge states. For this comparison we will focus on the most consistent feature of the *dI*/*dV* curves, namely the peak at negative sample voltage, which appears for both “cross” and “line” DB pairs.

**Figure 4:**
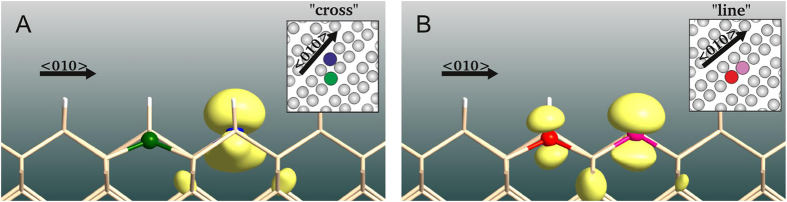
Iso-density (0.01 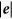 Å^3^) plot of the highest-energy unoccupied defect level associated with positively charged (+0) “cross” (left) and “line” (right) DB pairs. For the “line” structure the orbital extends to both DB sites but for the “cross” the orbital can be assigned unambiguously to one of the two DB sites.

**Figure 5:**
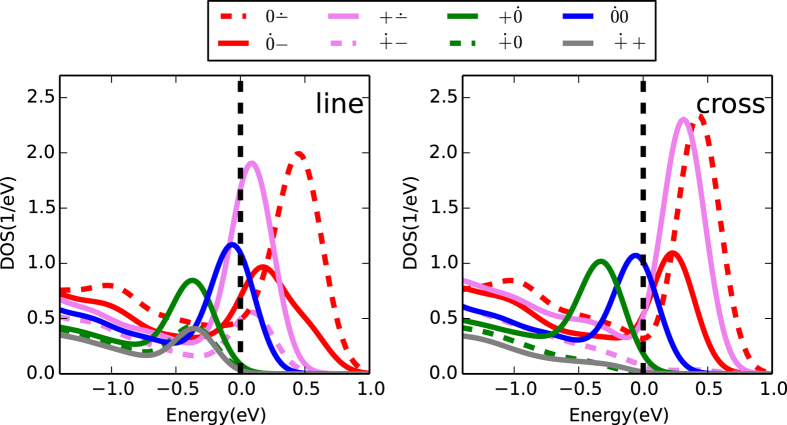
Projected density of states (PDOS) onto the Si atoms that form the DB pair. The PDOS are shown for all inequivalent sites and different charge states of the “cross” (left) and the “line” (right) pairs. A broadening of 0.2 eV was applied. Energies are given in relation to the valence band edge of the fully passivated surface, marked with a dashed line. The dot in the labels [*e.g.*, 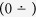] indicates which of the DB sites the PDOS belongs to.

In all the charged DB pairs, additional charge preferentially localizes to one of the DB sites, although this tendency is much stronger for the “cross” pair. This is exemplified in Fig. 4, where we represent the density associated with the highest-energy defect level of the positively charged DB pairs. Due to this localization we can introduce the notation (+0) to describe this configuration, where the two symbols refer to the charge state {−, 0, +} of each site. As mentioned above, for neutral DB pairs it was possible to stabilize both symmetric (00) and asymmetric (+−) configurations. In Tab. 1 we present the charges of different DB sites computed using Voronoi analysis^27^ for all the studied pairs and charge states. We see that the net charging of any DB site is far less than one electron - the gap states are only somewhat localized to the DB site and also extend deeply into the bulk. The PDOS of all inequivalent DB sites are shown in Fig. 5.

**Figure 6:**
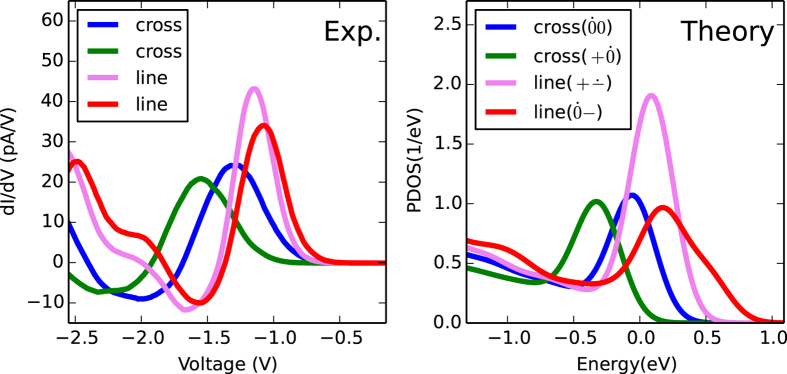
Comparison between experimental STS peaks (left) and the calculated PDOS of *selected* charge states (right). The colors have been chosen to emphasize the suggested match.

Combining the above considerations with a comparison of the relative position of the occupied PDOS peaks to that of the negative voltage STS peaks, we can attempt to identify the dominant charge state in the corresponding bias window (clearly below −0.5 V). Figure 6 shows our best match of the relative peak positions. The absolute positions are difficult to compare due to an unknown Fermi level and the effect of band bending and we therefore focus on the relative peak positions in our comparison between theory and experiment.

**Figure 7:**
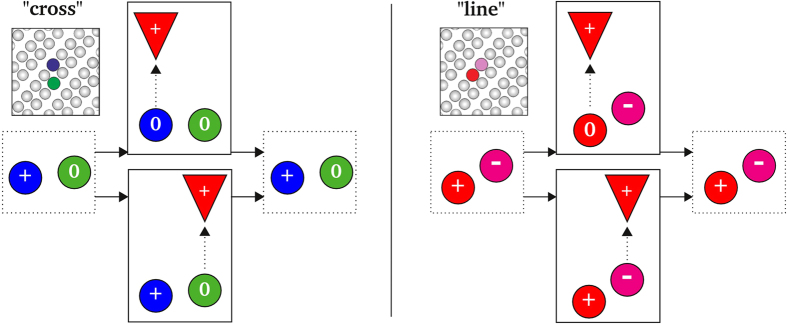
Our model to explain the measured STS spectra at negative sample voltage (corresponding to a positively charged tip). The basic ingredient is the assumption that in our p-doped Si(001):H substrate the transitions between positive and neutral charge states of the DBs can occur under moderate applied biases in the presence of the STM tip. In each panel we schematically show an initial state (left) whose dominant charge state can change under the influence of the STM tip (middle-top and bottom) and reaches a final state (right) after tip removal. (Left) The ground state of the “cross” DB pair is positively charged and corresponds to a (+0) configuration. However, the dominant charge state of the positive DB becomes neutral under the measuring conditions. (Right) The “line” DB pair is initially charge polarized, with a (+−) configuration. A similar positive → neutral transition takes place when the initially positive DB is measured. After measurement the DB pairs are left unmodified in agreement with experimental evidence.

For the “cross” DB pair our peak matching suggests that the negative voltage STS peak arises always from measuring a neutral DB in the presence of either a neutral or a positively charged DB (see Fig. 6). A scenario consistent with these observations is that the initial asymmetric pair-state has one neutral DB site and one positively charged DB site [(+0) configuration in our nomenclature]. Thus, that the ground state of the “cross” DB pair at our p-doped Si(001):H surface is positively charged. Then, during the recording of the STS spectra, as the sample voltage is ramped to more negative values, the positive DB site will change its dominant charge state to neutral when being measured (see Fig. 7). In contrast, with the relatively small applied voltages, the neutral DB site cannot change its charge state (to a negatively charged DB). Indeed, recent experiments^8^ for single DBs in Si(001):H indicate a large threshold sample voltage of 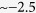 V for charging the DB with an extra electron in p-doped Si(001):H. Thus, our interpretation of the measured *dI*/*dV* spectra implies an easy transition (*i.e.*, using moderate applied voltages) between neutral and positive charge states for the DB at our Si(001):H surface. Notice, however, that this does not necessarily imply that such charge states are stable. All we need to invoke in our model is a modification of the *dominant* charge state of the DBs in the presence of the STM tip and under a sizable applied bias, *i.e.*, during measurement.

The origin of the shift of the *dI*/*dV* in the “cross” DB pair is then clear. We are always measuring the same type of DB (a neutral one), either in the presence of a positively charged 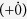 or a neutral companion 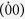. Therefore, the observed ~0.3 V shift is primarily of electrostatic origin, corresponding to the additional stabilization of one electron in a DB state provided by a positively charged neighbor. A similar mechanism of charge-state transitions (neutral → positive in this case) can also explain the similar shift observed experimentally for empty states (positive sample voltage in Fig. 1).

The matching in Fig. 6 for the “line” DB pair suggests that the measured *dI*/*dV* spectra correspond to the theoretical PDOS calculated for the 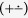 and 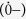 configurations. This could occur, e.g., if the initial and final state of this structure are globally neutral but charge polarized, corresponding to a positively charged DB and a negatively charged companion (+−), respectively (see Fig. 7). Unfortunately, this is in conflict with the data in Table 1 that point towards a symmetric ground state of the “line” pair. However, the energy difference between symmetric and asymmetric structures is rather small in the case of the “line” DB pair (70 meV). Thus, taking into account the uncertainty associated with the use of a semilocal density functional, as well as that related to the use of large supercells (notice that this small energy difference is computed in a cell of 430 atoms, with full relaxations), it is difficult to consider the energy ordering in Table 1 as a conclusive argument for rule out the asymmetric (+−) configuration as the initial state for the “line” pair. Furthermore, with such a small energy difference, the structure of the DB pair might be influenced by the presence of the STM tip already at much lower biases than those necessary to produce the change of charge state, thus playing a role in determining the “initial” state for the process depicted in Fig. 7. In any case, since an asymmetric initial state is required to understand the measured STS, in our p-doped sample we are left with the (+−) and (+0) states as the most plausible choices. Although we cannot completely rule out the (+0) charge state as a carrier of the asymmetry, in order to produce a good matching with the measured STS, (+−) is the best choice as a initial/final state. In particular, starting from (+−) we can have an excellent matching with the experimental results just invoking the neutralization of the positively charged DB under a moderate negative bias, i.e., a positive → neutral transition of the DB.

The proposed scenario provides good correspondence between the calculated and measured relative positions of the filled-state peaks in STS. However, our central hypothesis, namely that the charge state of one DB in a pair can be modified without affecting the state of the companion DB (situated just a few ångströms away) may seem quite surprising at first and, thus, requires further discussion. Several experimental investigations to date have proven that the charge state of a DB site can be changed during STS/STM measurements^4^,^8^,^20^,^31^. Two effects are usually invoked to justify this observation: (i) the possibility that the current through the localized electronic state in the DB determines the steady-state charge state of the DB^32^; (ii) the effect of band bending, that shifts the relative positions of the DBs in the surface and the Fermi energy of the sample^8^,^20^. The first mechanism depends on the spatial distribution of the tunneling current density and, thus, can be expected to be very local. However, the currents used here (in the 10 pA range) are substantially smaller than those which have been observed to influence the charge state of isolated DBs in ref. 32 (above 100 pA in order to charge negatively an isolated DB in n-doped Si(001):H). Therefore, the actual importance of this mechanism under our experimental conditions is not clear.

In contrast to the tunneling current, the lateral variations of the tip-induced band bending are usually considered to take place on the scale of the curvature radius of the STM tip^32^ and, therefore, larger than the distances between the pair of DBs considered here. This would indicate that the band-bending effects should be similar for both DBs in our pairs. Of course, the high resolution of our STM images indicates that the apex of the tip has atomic dimensions. This makes conceivable that the tip-induced voltage profile along the surface changes in the length-scale of the tip-surface separation, providing a way to justify its different effect in close-spaced DB pairs.

One particularly important point is that our model only requires neutral↔positive transitions, which involve the charging/decharging of a level located close to the top valence band. Therefore, this process can take place at relatively small applied biases and it is plausible that can be affected by small differences in the level position induced by moving the tip a few ångströms away from a given DB site.

## Conclusions

We have demonstrated that STS spectra of close-spaced DB pairs at our p-doped Si(001):H surface exhibit a remarkable resemblance but appear shifted relative to each other by a constant energy that depends on the relative positions of those DBs. This spontaneous and robust asymmetry persists over extended periods of time—it can be repeatedly probed by STS measurement without being changed. By comparing the STS data to the calculated position of the electronic states in DFT calculations for different charge states of the defects, we have proposed a model that explains the observed behavior, reproducing the size of the observed shifts. First, our calculations indicate that the initial state of the studied DB pairs is always characterized by an asymmetric distribution of the electronic charge. In particular, in our p-doped Si(001):H substrate one of the DB in the pairs always seems to be positively charged. Second, we assume that while being measured (*i.e.*, in presence of a STM tip and with an applied sample voltage in the range of −0.5 to −1 V) the positively charged DB can undergo a transition to a neutral charge state. This transition is reverted as soon as the measurement is finished and the DB pair is left in its initial (asymmetric) configuration. These two ingredients are sufficient to account for the observed spectroscopic data and provide a framework to understand this type of experiments.

In the case of the “cross” DB pair, where the electrons are fairly localized to a particular DB site, the observed shift of ~0.3 V in the STS can be interpreted as an approximate measurement of the electrostatic interaction between the neighboring DB sites. However, for the “line” structure, where the electronic states tend to be more delocalized among both DB sites, such simple interpretation can be questioned.

An interesting consequence of our interpretation of the experimental information is that it indicates the possibility of controlling the charge state of individual DBs in complex structures, even if there are neighboring DBs in close proximity. This is an important observation in the context of the use of DB structures as active components in future atomic-scale electronic circuits^18^.

## Additional Information

**How to cite this article**: Engelund, M. *et al.* Tunneling spectroscopy of close-spaced dangling-bond pairs in Si(001):H. *Sci. Rep.* **5**, 14496; doi: 10.1038/srep14496 (2015).

## Figures and Tables

**Table 1:** Total energies of locally neutral and charge-polarized states for neutral DB pairs.

	line (eV)	cross (eV)
00	0.00	0.15
+−	0.07	0.31

The (00) state correspond anti-aligned spins.

**Table 2:** Voronoi partial charges at the DB sites for diffetential nominal charge-states of the DB pairs.

	line (Q)		cross (Q)	
	site 1	site 2	site 1	site 2
0−	−0.01	−0.11	−0.01	−0.11
00	0.00	0.00	0.00	0.00
+−	0.07	−0.07	0.13	−0.10
+0	0.10	0.04	0.13	0.01
++	0.10	0.10	0.13	0.13

As can be appreciated by the low partial charges, the DB states are not strictly localized to the DB sites in the surface, but have significant tails into the bulk region.
